# A novel pathogenic *CDH3* variant underlying heredity hypotrichosis simplex detected by whole-exome sequencing (WES)—a case report

**DOI:** 10.1101/mcs.a006225

**Published:** 2022-08

**Authors:** Ayat Kadhi, Lamiaa Hamie, Christel Tamer, Georges Nemer, Mazen Kurban

**Affiliations:** 1 Division of Genomics and Translational Biomedicine, College of Health and Life Sciences, Hamad Bin Khalifa University, Doha 3411, Qatar;; 2 Sidra Medicine, Doha 26999, Qatar;; 3 Department of Dermatology, Faculty of Medicine, American University of Beirut, Beirut 110236, Lebanon;; 4 Department of Radiology, American University of Beirut, Beirut 110236, Lebanon;; 5 Department of Biochemistry and Molecular Genetics, Faculty of Medicine, American University of Beirut, Beirut 110236, Lebanon

**Keywords:** progressive hypotrichosis

## Abstract

Heredity hypotrichosis simplex (HHS) is a rare nonsyndromic disease form of hypotrichosis simplex (HS) characterized by progressive hair follicle (HF) miniaturization. It is usually inherited in an autosomal dominant manner. The differential diagnosis of HHS and the treatments remain challenging despite recent advancement. In this report, we describe a 19-yr-old female affected with HHS alongside most of her family members. Whole-exome sequencing (WES) was performed for some of the family members to unravel the culprit gene involved in HHS phenotype and ascertain the dermatological examination that was done to classify the phenotypes of the disease. A novel pathogenic variant in the *CDH3* gene (p.Ser223GlyfsTer4) was identified as a plausible disease-causing variant for HHS. This is the first report to associate *CDH3* variants with a HHS phenotype without macular degeneration using WES. WES is an important tool for genotype–phenotype correlation, precision in diagnosis, and in-depth understanding of the disease mechanisms, leading to possible novel therapeutic targets treatment and better patient outcomes.

## INTRODUCTION

Heredity hypotrichosis simplex (HHS) is a form of nonsyndromic hypotrichosis characterized by progressive hair shaft thinning leading to diffused hair loss. Hypotrichosis's prevalence is still unknown, but it is very rare and has been reported mainly in Arab, Danish, and Pakistani families (Betz et al. 2000; Levy-Nissenbaum et al. 2003; Shimomura et al. 2010a). The onset of the disease usually begins in early childhood and progress with age. It can be divided into scalp-limited form (HYPT2; OMIM #146520) and the generalized form (HYPT1; OMIM #605389). The disease can be inherited either in an autosomal dominant or autosomal recessive manner and affects men and women equally (Toribio and Quinones 1974; Bentley-Phillips and Grace 1979; Carvalho et al. 2006; Schaffer et al. 2006; Shimomura et al. 2010a). Variants in genes such as corneodesmosin (*CDSN*) and small nuclear ribonucleoprotein polypeptide E (*SNRPE*) usually result in hypotrichosis as an isolated finding, whereas variants in *CDH3* gene lead to hypotrichosis as a part of a syndrome like hypotrichosis simplex with juvenile macular dystrophy (HJMD; OMIM #601553) or ectodermal dysplasia, ectrodactyly and macular dystrophy (EEM; OMIM #225280) (Indelman et al. 2005; Kjaer et al. 2005; Hua et al. 2021; Pan et al. 2021).

Paradoxically, we present the clinical characteristics of a case affected with generalized nonsyndromic HHS associated with a novel *CDH3* gene variant in a nonconsanguineous Lebanese family using WES, which have not been described in the literature.

## RESULTS

### Clinical Presentation

**Figure 1.**
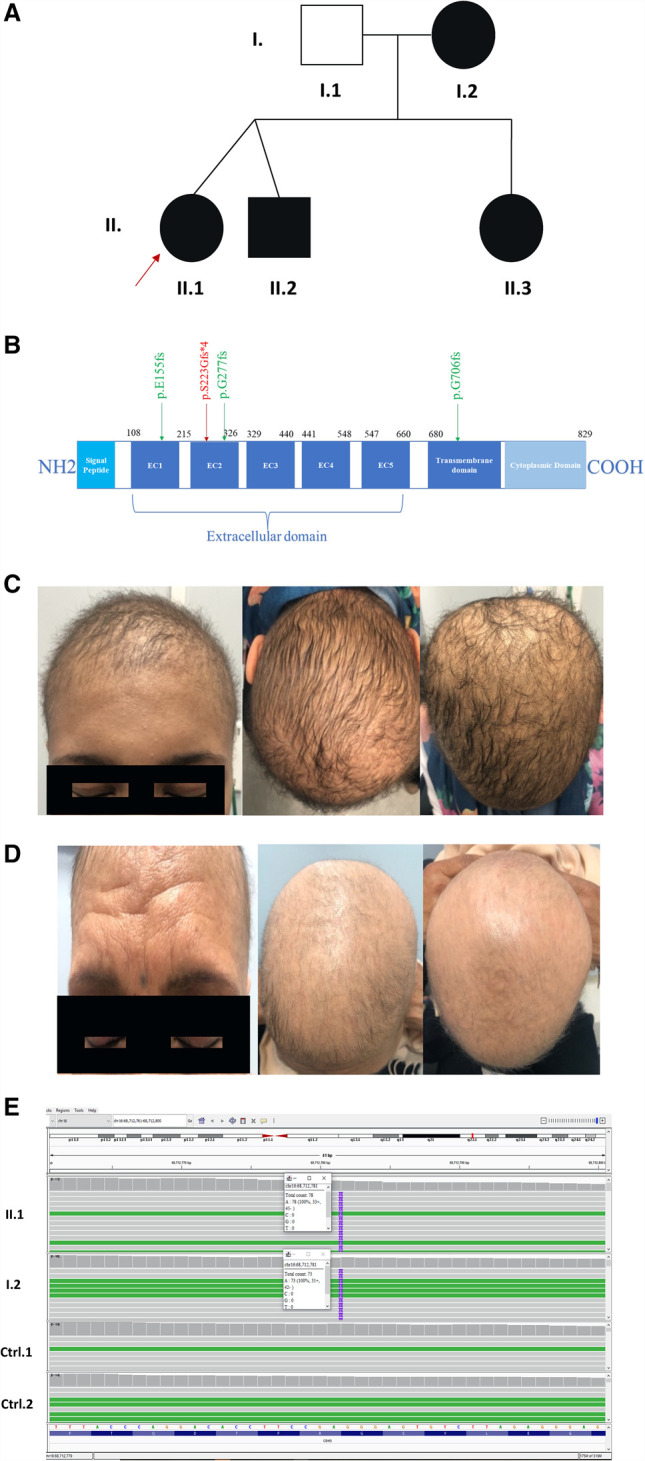
*CDH3* p.Ser223GlyfsTer4 a frameshift variant that segregates with heredity hypotrichosis simplex. (*A*) The pedigree of the family, nonconsanguineous marriage. All members are diagnosed with HHS (highlighted by black color), except the father (highlighted with white color). The proband is highlighted with red arrowhead. (*B*) CDH3 protein structure and domains (829 amino acid). The variant detected highlighted in red in the extracellular domain 2; only frameshifts variants and associated with HJMD from previous reports are highlighted in green. (*C*) Hypotrichosis features presented in the patient. (*D*) Hypotrichosis features presented in the mother. (*E*) IGV visualization of *CDH3*; a two G nucleotide insertion leading to variant change in the homozygous form for the patient (II.1 and the mother I.2) and absent in controls (*bottom* panels).

The proband II.1 is a 19-yr-old female, presented with complete scalp alopecia (nonscarring form) at birth. Later, she presented with short villus hair over the scalp, short eyelashes, normal eyebrows, and her hair density was significantly reduced on the forearms, her mother had similar physical phenotype (Fig. 1B,C). The detailed physical examinations are summarized in Table 1. Her mother (I.2) had similar physical phenotype, but her hair density was less (Fig. 1C,D). Both were diagnosed with hypotrichosis simplex by a dermatologist, skin biopsy was taken from the scalp in order to confirm the diagnosis. The proband II.1 complete blood count (CBC), calcium levels, and thyroid hormones were within normal ranges. She only had an elevated parathyroid hormone (PTH) and slightly low zinc levels, which is not significant to the disease prognosis (Table 2). None of the family members did show any sign of macular degeneration upon full field electroretinography (ERG) analyses at different time intervals. The affected family members were advised to repeat the ophthalmological exam every 2 yr.

**Table 1.** Clinical findings

Hypotrichosis simplex clinical features^a^	Proband II.1	Mother I.2	Father I.1
Sparse eyebrows	No	No	No
Sparse eyelashes	Yes	Yes	No
Sparse, thin, and short scalp hair	Yes	Yes	No
Sparse body hair	Yes	Yes	No
Sparse axillary hair	Yes	Yes	No
Sparse pubic hair	Yes	Yes	No
Hypotrichosis, generalized	Yes	Yes	No
Normal scalp hair density at birth	No	No	No
Hypotrichosis, generalized	Yes	Yes	No
Normal skin	Yes	Yes	Yes
Normal sweat glands	Yes	Yes	Yes
Normal scalp (skin)	Yes	Yes	Yes
Normal nails	Yes	Yes	Yes
Normal teeth	Yes	Yes	Yes
**Novel clinical features**			
Hyperparathyroidism	Yes	Unknown	Unknown
Low zinc levels	Yes	Unknown	Unknown

^a^The list of clinical features is based on OMIM clinical synopsis (HYPOTRICHOSIS 1; HYPT1 #605389) Clinical Synopsis - #605389—HYPOTRICHOSIS 1; HYPT1—OMIM.

**Table 2.** Blood test results for Proband II.1

Test	Result	Reference range
Hematology		
WBC (c/uL)	6100	4600–10,200
Neutrophils bands (%)	0	0–2
Neutrophil segmented (%)	59	40–65
Lymphocytes (%)	24	25–40
Monocytes (%)	7	02-Aug
Eosinophils (%)	10^a^	0–4
Basophils (%)	0	0–1
RBC (M/dL)	4.77	4–5.5
HGB (g/dL)	13.6	Dec-16
HCT (%)	40.1	37–46
MCV (g/dL)	84	80–99
MCH (g/dL)	29	27–32
MCHC (g/dL)	33	31–36
Platelets (c/µL)	21.000	150,000–450,000
Chemistry		
Calcium S. (mg/dL)	9.3	8.5–10.5
Zinc (µg/dL)	61^a^	70–114
Endocrinology		
Parathormone (PTH) (pg/mL)	120^a^	12–72
TSH (ultrasensitive) µIU/mL	4.54	0.3–4.9
Free T4 (ng/dL)	1.02	0.8–1.9

^a^Exceed limits.

In this case of nonconsanguineous marriage, the family history was remarkable with HHS, along with the indexed patient, two of the siblings and her mother was affected. The father was the only unaffected member (Fig. 1A).

### Genomic Analyses and Variant Interpretation

To unveil the genetic basis of the phenotype and ascertain the clinical diagnosis, we performed whole-exome sequencing (WES) for the patient and her mother. WES provides a more convenient diagnosis tool by avoiding painful diagnosis measures such as skin biopsies (Trujillano et al. 2017). Unfortunately, not all members of the family consented to DNA sequencing. The results of the first proband and the mother yielded 79,007 variants per sample before applying stringent filter and 603 variants afterwards (see Methods). Alongside the stringent filter, the variants were classified and ranked based on the clinical data findings while assuming an autosomal pseudodominant mode of inheritance and adhering to the American College of Medical Genetics and Genomics (ACMG) standards and guidelines. A novel homozygous variant (Table 3) located on 16q22.1 mapping to exon 6 (of 16) of the *CDH3* gene (exon level c.663_664insGG and genome level g.42690_42691insGG) was detected in the proband and her mother resulting from an insertion of two cytosine nucleotides (Fig. 1E). The p.Ser233GlyfsTer4 (NM_001793.4) variant maps to the extracellular domain 2 of the protein leading to a premature stop codon (Fig. 1B). The variant is predicted to be pathogenic (https://varsome.com/variant/hg19/Chr16%3A68712781%3AA%3AAGG?) according to the ACMG classifications (Li and Wang 2017) and has a deleterious effect with a tree vote of 200|0 (del|benign) that leads to a nonsense-mediated decay (NMD) according to mutation taster prediction tool (https://www.genecascade.org/MT2021/MutationTaster102.cgi?start_insdel=42690&bases_inserted=GG&sequence_type=gDNA&end_insdel=42691&transcript_stable_id_text=ENST00000429102&alteration_name=16:68712781A%3EAGG_1_ENST00000429102) (Steinhaus et al. 2021). The variant was absent from the genomes/exomes databases in gnomAD (Gudmundsson et al. 2021), ExAC (Lek et al. 2016), 1000G (1000 Genomes Project Consortium et al. 2015), and 300 Lebanese in-house exomes.

**Table 3.** Genomic findings

Patients	Gene	Chromosome	Zygosity	HGVS DNA reference	HGVS protein reference	Coding impact	Predicted effect (ACMG/mutation taster)	MAF (gnomAD)	MAF (Lebanese exome population)
I.2, II 1	CDH3	Chr16:687127 81A > AGG GRCh37/hg19	Homozygous	c.665_666dupGG	(p.Ser223GlyfsTer4)	Frameshift	Pathogenic (PVS1)/deleterious (200|0)	0/251,486	0/300

## DISCUSSION

The hair follicle is a complex structure within the skin that regenerates regularly in the hair cycle form. Many genes have been identified to be expressed in this milieu providing a specific-molecular signature that distinguishes it from other structures (Schmidt-Ullrich and Paus 2005). Previous studies have reported many casual genes associated with heredity hypotrichosis simplex such as *LSS*, *CDSN*, and *APCDD1* (Shimomura et al. 2010a; Pasternack et al. 2013; Peled et al. 2020), while other studies highlighted that *CDH3* variants cause hypotrichosis simplex with juvenile macular dystrophy (HJMD; OMIM #601553) that is manifested later in life (Sprecher et al. 2001; Saeidian et al. 2019; Schauren et al. 2020; Ahmed et al. 2021). Paradoxically, we identified *CDH3* as a casual gene for HHS and in the absence of macular degradation in both the patient and her mother. Neither of them carried any casual genes associated with HHS.

*CDH3* encodes P-cadherin, which plays a key role in cell signaling regulation, major intracellular processes, and most importantly cell–cell adhesion through intracellular junctions. P-cadherin is the only cadherin expressed in the innermost hair matrix (IHM) and epithelial cells during hair morphogenesis but is poorly expressed in the epidermal cells and skin appendages (Shimomura et al. 2008; Samuelov et al. 2012).

In contrast, E-cadherin encoded by *CDH1* is expressed abundantly in the epidermal cells and skin appendages (Hirai et al. 1989; Fujita et al. 1992). These findings could explain the sparse short hair formation and the absence of any skin phenotype as the variant in *CDH3* gene that affects P-cadherins only. In published genetic studies, identical variants in *CDH3* were linked to various phenotypes in different populations. For example, the variant (p.G277Afs*20) was associated with both HJMD and EEM (Indelman et al. 2003; Kjaer et al. 2005). This phenotypic heterogeneity suggests the possibility of the presence of a modifier gene at the same locus or on different chromosomes (Kjaer et al. 2005). It has been suggested that *CDH1* might be one of the modifier genes contributing to partial functional compensation as both encoded cadherin proteins (P and E) have some redundant functions. *CDH1* residues closely to *CDH3* (38 kb) and is expected to cosegregate genetically unless rare recombination events occur (Shimomura et al. 2010b). Both P-cadherin and E-cadherin are coexpressed in the apical epidermal ridge in mice during limb development (Shimomura et al. 2008). The fact that E-cadherin is a gene modifier could explain how some patients with deleterious P-cadherin variants have normal limb development. In our case, the phenotypes observed in the indexed patient and her mother is slightly different. However, we did not find any variant in *CDH1*, but we hypothesize that age and/or epigenetic reprogramming might explain this difference.

Finally, other modifier genes might explain the variable phenotypes associated with *CDH3* variants in different patients. This is the case of two unrelated consanguineous Pakistani families that have been reported to have heredity hypotrichosis (scalp limited or generalized) as an isolated finding, a very similar phenotype to our case. The two families had pathogenic variants in *CDH3* along with variants in a novel locus on chromosome 12q21.2-q22. This digenic inheritance expand the genetic heterogeneity of hypotrichosis and the possibility of a modifier gene on chromosome 12 might explain the unique phenotype in their case (Basit et al. 2011). The fact that this variant does not lead to macular degeneration could be explained by the location of the variant itself within the protein as total loss of function was previously linked to a severe phenotype. One would hypothesize that the residual amino-terminal activity is sufficient to prevent such a phenotype.

## SUMMARY

In conclusion, this is the first report highlighting a novel frameshift variant in *CDH3* to be associated with heredity hypotrichosis simplex without macular degeneration based on the clinical and familial segregation data.

## METHODS

### Research Participants and Clinical Data

The recruitment of this family was done at the Genodermatoses’ unit at the department of dermatology at the American University of Beirut Medical Center (AUBMC). Clinical phenotypes were provided by the referring physician.

An amount of 5 mL of peripheral blood was collected from the participating patients and kept at 4°C. DNA was extracted using the QIAamp Blood Midi kit (QIAGEN Sciences, Inc.), as per the manufacturer's instructions. DNA quantification was performed on a NanoDrop (Thermo Fisher Scientific, Inc.) at the molecular core facility at AUB. Five micrograms of coded DNA samples was shipped to Macrogen, where exome sequencing was performed.

### Whole-Exome Sequencing and Bioinformatic Analysis

Whole-exome sequencing (WES) was performed for the proband and the mother by Macrogen Laboratory (https://dna.macrogen.com/). One-hundred 1-bp (pair-ended) reads were sequenced using Illumina NovaSeq6000 platform. The library preparation was performed according to the manufacturer's protocol. In brief, using random fragmentation of the DNA, the library was prepared followed by 5′ and 3′ adapter ligation and tagmentation. After that, adapter-ligated fragments were amplified by polymerase chain reaction (PCR) and gel purification, and the library was loaded into a flow cell. The fragments were captured in this flow cell on a lawn of surface-bound oligos complementary to the library adapters for cluster generation. Later, each fragment was amplified using bridge amplification into distinctive, clonal clusters. By that, cluster generation was complete, and the templates were ready for sequencing. The data sequenced were converted to raw data, base calling was done using Illumina sequencer, and raw images were generated using RTA (Real Time Analysis) software. Finally, using Illumina package bcl2fastq, the BCL (base calls) binary was converted into FASTQ (paired-end reads) for the analysis. The Phred quality value was around +33, which means that the base call accuracy was 99.9%. More information about the quality of the data generated is summarized in Table 4.

**Table 4.** Sequencing data quality

Sample	Total bases	Read count	GC (%)	AT (%)	Q20 (%)	Q30 (%)
Proband II.1	5,896,463,628	39,049,428	52.31	47.69	97.60	93.58
Mother I.2	6,045,972,050	40,039,550	51.93	48.07	97.76	93.85

### Exome Analysis, Annotation, and Filter

The FASTQ files were mapped to Human GRCh37/hg19 reference assembly using CLC Genomics Workbench (version 20.0.4). Failed reads and broken reads were removed. Minimum coverage of 10 minimum count of two reads, and minimum frequency of 35% were included. Basic variant parameter ploidy was set at 2. For reference masking, positions with coverage above 100,000 were ignored. For each sample, Binary Alignment Map (BAM) and Variant Call Format (VCF) files were generated that included all the variants using CLC Genomics Workbench (version 20.0.4).

Variant calling and annotation were done by uploading VCF files to Illumina Variant Studio 3.0. Annotation was based on dbSNP, ClinVar, and the 1000 Genomes Project. After that, a stringent filter was applied for all the samples where we included variants with read depth of >20, minor allele frequency (MAF) of <5% based on the 1000 Genomes Project, ExAC, and EVS Max. The variant type included was single nucleotide variant (SNV), insertion, and deletions, the variant types nonsense, missense, frameshift, splice. Along the stringent filter, every patient was filtered against more than 300 Lebanese in-house exomes to exclude any repetitive variant. We looked for the known genes that cause HHS but could not find any; then we looked for novel variants with high pathogenicity scores by using in silico prediction tools to further confirm the potential variant. Databases such as American College of Medical Genetics and Genomics (ACMG) classification and mutation taster, The Genome Aggregation Database (gnomAD) Genomes/Exomes coverage, and PhastCons and PhyloP were used. All of the potential variants were then manually curated with literature using Pubmed and Online Mendelian Inheritance in Man (OMIM), leading to the identification of the present variant. BAM files were uploaded to the Integrative Genomics Viewer (IGV; Broad Institute) as a high-performance visualization tool for genomic annotations and correct calling.

## ADDITIONAL INFORMATION

### Data Deposition and Access

Exome-sequencing data have been deposited in the National Center for Biotechnology Information (NCBI) Sequence Read Archive (SRA) (http://www.ncbi.nlm.nih.gov/sra/) under accession numbers SRR19632076 and SRR19632075. The *CDH3* c.665_666insGG, p.Ser233GlyfsTer4 variant has been deposited to ClinVar (http://www.ncbi.nlm.nih.gov/clinvar/) under accession number SCV002520327.

### Ethics Statement

Institutional Review Board of the American University of Beirut Medical Center approval and written informed consent were obtained from the research participants patients, or their parents (if minors).

### Acknowledgments

We thank the members of the family for their contribution to this research project.

### Author Contributions

L.H., C.T., and M.K. recruited the family and performed the clinical workup. A.K. did the exome sequencing and interpretation of results, in addition to writing the first draft of the manuscript. M.K. and G.N. conceived the work, and all authors participated in the final writing of the manuscript.

### Competing Interest Statement

The authors have declared no competing interest.

### Referees

Sangeeta Ghuwalewala; Nesrin R. Mwafi
